# Full-genome sequences of hepatitis B virus subgenotype D3 isolates from the Brazilian Amazon Region

**DOI:** 10.1590/0074-02760140426

**Published:** 2015-02

**Authors:** Natália Spitz, Francisco CA Mello, Natalia Motta Araujo

**Affiliations:** 1 Laboratório de Virologia Molecular, Instituto Oswaldo Cruz-Fiocruz, Rio de Janeiro, RJ, Brasil; 2 Departamento de Microbiologia e Parasitologia, Universidade Federal Fluminense, Niterói, RJ, Brasil

**Keywords:** HBV, complete genome, amino acid residues, genetic distance

## Abstract

The Brazilian Amazon Region is a highly endemic area for hepatitis B virus (HBV). However, little is known regarding the genetic variability of the strains circulating in this geographical region. Here, we describe the first full-length genomes of HBV isolated in the Brazilian Amazon Region; these genomes are also the first complete HBV subgenotype D3 genomes reported for Brazil. The genomes of the five Brazilian isolates were all 3,182 base pairs in length and the isolates were classified as belonging to subgenotype D3, subtypes ayw2 (n = 3) and ayw3 (n = 2). Phylogenetic analysis suggested that the Brazilian sequences are not likely to be closely related to European D3 sequences. Such results will contribute to further epidemiological and evolutionary studies of HBV.

Hepatitis B virus (HBV) infection is one of the major causes of chronic liver diseases, including cirrhosis and hepatocellular carcinoma and affects over 240 million people worldwide (WHO 2014 ). HBV contains a partially double-stranded DNA genome approximately 3.2 kb in length. Eight confirmed (A-H) and two tentative (I and J) genotypes have been identified based on a nucleotide divergence of more than 8% for the complete genome (Araujo et al. 2011). Much diversity within genotypes exists, leading to the division of some genotypes into subgenotypes (Shi et al. 2013). Both HBV genotypes and subgenotypes have different geographic distributions (Kramvis et al. 2005) and have increasingly been associated with differences in transmission routes, disease progression, responses to antiviral therapies and clinical outcomes (McMahon 2009, Lin & Kao 2011 ).

Genotype D has a worldwide distribution, but is found primarily in the Mediterranean area, Eastern Europe and a region spanning from the Near East to India. It has been associated with a high risk of disease progression and a poor clinical outcome (Lin & Kao 2011). Nine subgenotypes (D1-D9) have so far been described (Ghosh et al. 2013, Shi et al. 2013). However, subgenotypes D3 and D6 were recently reclassified as a single subgenotype, D3 (Shi et al. 2013, Yousif & Kramvis 2013). This latter subgenotype has been found primarily in Northern America, Europe, South Africa and Indonesia (Yousif & Kramvis 2013).

Brazil has a highly admixed population with Caucasian, Amerindian and African origins. Genotypes A, D and F circulate among Brazilian HBV carriers (Mello et al. 2007). Genotype D has been found in all five Brazilian geographic regions (Mello et al. 2007), with a predominance of the D3 subgenotype observed countrywide (N Spitz et al., unpublished observations). It has been proposed that the D genotype in Brazil has a European origin, because the highest rates of genotype D are found in the southern region, where an influx of immigrants from Central Europe, especially Germany and Italy, has occurred (Mello et al. 2007, Bertolini et al. 2012). The Brazilian Amazon Region is a highly endemic area for HBV (Viana et al. 2005) and 24.4% of the HBV strains isolated in this region have been shown to be of genotype D (Mello et al. 2007). However, little is known about the genetic variability of the HBV strains circulating in the Brazilian Amazon Region and no complete genome sequences from this region have been described to date. In addition, few Brazilian HBV complete genome sequences are available in the GenBank database and this has limited the contribution of Brazilian isolates to molecular epidemiological and phylogenetic studies of HBV.

In this paper, we describe the first full-length genomes of HBV isolated in the Brazilian Amazon Region; moreover, these genomes are the first complete genomes of HBV subgenotype D3 reported in Brazil.

Complete genome sequences were obtained for HBV isolates from five HBsAg-positive blood donors residing in the states of Amapá (sequences BR2, BR4 and BR6) and Amazonas (sequences BR14 and BR40). This study was approved by the Brazilian Ethical Committee for Medical Research (registration 9604/2004). HBV DNA was extracted from 0.2 mL of serum using a High Pure Viral Nucleic Acid kit (Roche Diagnostics, Germany) and full-length HBV genomes were amplified as described previously (Gunther et al. 1995). HBV nucleotide sequences were determined using a BigDye Terminator kit (Applied Biosystems, USA) and sequencing reactions were analysed on an ABI3730 automated sequencer (Applied Biosystems). The nucleotide sequences reported here were deposited in the GenBank database under accessions KP090177-KP090181. Phylogenetic analysis was conducted using MEGA software v.6 (Tamura et al. 2013). Phylogenetic trees of the HBV full-genome sequences were obtained using the neighbour-joining method (1,000 bootstrap replicates) and mean genetic distances were estimated using the Kimura two-parameter model. Bootscan analysis software (SimPlot v.3.5.1) was used to identify intra and intergenotypic recombination (Lole et al. 1999).

All five complete HBV genome sequences were 3,182 base pairs in length and contained the canonical HBV overlapping open reading frames for C (HBe, 639 nt; HBc, 552 nt), X (HBx, 465 nt), PreS/S (LHBs, 1170 nt; MHBs, 846 nt; SHBs, 681 nt) and P (Pol, 2499 nt). The deduced amino acid sequences of the small S protein of the BR6, BR14 and BR40 isolates contained R, P and K residues at positions 122, 127 and 160, respectively, corresponding to the ayw2 serological type (subtype); BR2 and BR4 instead had the ayw3 subtype (R122, T127, K160). Neither in-phase deletions or insertions nor the important mutations G1896A (PreC), rtM204V (lamivudine resistance mutation), A1762T and G1764A (in the basal core promoter) were detected in the sequences and no evidence of recombination was observed in the sequences, as well.

By phylogenetic analysis, it was demonstrated that the five genomes clustered together with subgenotype D3 sequences from other countries (Figure). However, Brazilian D3 sequences did not produce a single cluster, suggesting that this subgenotype may have been introduced into the country multiple times. Moreover, the Brazilian sequences seemed not to be closely related to European sequences (Figure). It would be useful to investigate the lack of relatedness between Brazilian and European D3 sequences in further studies focusing on the phylogeography of HBV in Brazil.

**Figure:**
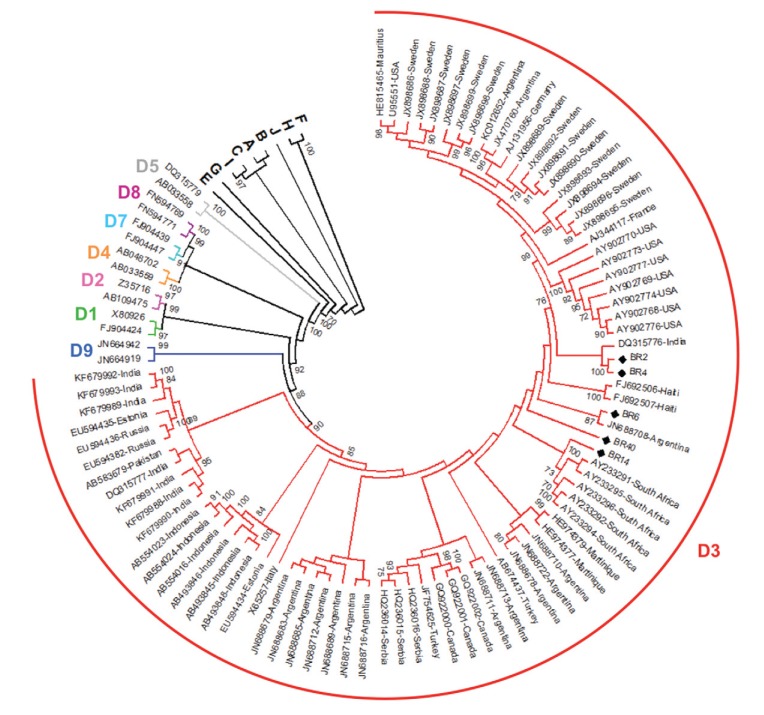
Phylogenetic analysis of HBV sequences using the neighbour-joining method. GenBank accessions for the reference sequences are: genotype A, AY233278; B, D00329; C, AB112066; E, X75664; F, X69798; G, AB056513; H, AY090454; I, FJ023660; J, AB486012. Genotype D reference sequences are indicated by their accession numbers. Genotype D3 sequences are indicated by their accession numbers followed by the name of the origin country. The sequences generated in this study are denoted BR, followed by the sample number and are identified with the symbol . Values at internal nodes indicate percentages of 1,000 bootstrap replicates that support the branch.

The deduced amino acid sequences of the viral polymerase, X, PreS2 and S proteins of the five subgenotype D3 isolates from Brazil were compared (Table). Variations in the amino acid residues between the ayw2 and ayw3 isolates, in addition to the P127T substitution in the S gene, were observed in several positions throughout the genome: polymerase, residue sp116 (L or F), sp142 (N or S), rt53 (N or D), rt135 (S or Y), rt266 (V or I), rh19 (A or V) and rh88 (V or I); X, residue 17 (C or Y) and 26 (R or C); PreS2, 31 (T or A) and S, 125 (T or M). A larger number of complete subgenotype D3 genomes from Brazil is needed to confirm such variations in the predicted amino acid residues between the ayw2 and ayw3 isolates, as these variations may have implications for disease pathogenesis and progression.

**TABLE:** Comparison of the amino acid residues encoded by the different subgenomic regions of Brazilian HBV subgenotype D3 isolates - correlation with the sample subtype

		Polymerase								X			PreS2		S	
Sample	Subtype	sp116	sp142	rt53	rt135	rt266	rh19	rh88		17	26		31		125	127^*a*^
BR2	ayw3	F	S	D	Y	I	V	I		Y	C		A		M	T
BR4	ayw3	F	S	D	Y	I	V	I		Y	C		A		M	T
BR6	ayw2	L	N	N	S	V	A	V		C	R		T		T	P
BR14	ayw2	L	N	N	S	V	A	V		C	R		T		T	P
BR40	ayw2	L	N	N	S	V	A	V		C	R		T		T	P

a: amino acid residue that distinguishes ayw2 from ayw3.

The genetic information provided here will help us to understand better the evolutionary behaviours of HBV subgenotype D3 strains circulating in the Brazilian Amazon Region and to trace the spread of disease due to HBV in this part of the world.
